# Association Between Chronic Diseases and Frailty in a Sample of Older Greek Inpatients

**DOI:** 10.7759/cureus.58568

**Published:** 2024-04-18

**Authors:** Andreas Kyvetos, Eleni Kyritsi, Ioannis Vrettos, Panagiota Voukelatou, Anastasia D Manoli, Elektra Papadopoulou, Odysseas F Katsaros, Konstantinos Toutouzas

**Affiliations:** 1 Second Department of Internal Medicine, General and Oncology Hospital of Kifissia “Agioi Anargyroi”, Athens, GRC; 2 First Department of Cardiology, Medical School, Hippokration General Hospital of Athens, National and Kapodistrian University of Athens, Athens, GRC; 3 Department of Pediatric Medicine, Pendelis General Children’s Hospital, Athens, GRC; 4 Department of Cardiology, Evangelismos General Hospital, Athens, GRC

**Keywords:** clinical frailty scale, older-aged patients, aging, cardio vascular disease, frailty syndrome

## Abstract

Introduction: Previous reports have associated frailty with the existence of various chronic diseases. Especially for cardiovascular diseases, this relationship seems to be bidirectional as common pathophysiological mechanisms lead to the progression of both diseases and frailty. The study aimed to examine the relationship between chronic diseases and frailty in a sample of older Greek inpatients

Methodology: In 457 consecutively admitted older patients (226, 49.5% females), the median age was 82 years (interquartile range [IQR] 75-89), and demographic factors, medical history, cause of admission, and the degree of frailty assessed with the Clinical Frailty Scale were recorded. The level of frailty was calculated for the pre-hospital status of the patients. Parametric tests and logistic regression analysis were applied to identify diseases independently associated with frailty.

Results: Using the scale, 277 patients (60.6%) were classified as frail and 180 as non-frail (39.4%). In univariate analysis, frail patients were more likely to have respiratory disease, dementia, Parkinson's disease, chronic kidney disease (CKD), atrial fibrillation (AFIB), neoplastic disease, depression, stroke, heart failure (HF), and coronary artery disease. In binomial regression analysis, the diseases that were statistically significantly associated with frailty were respiratory diseases (*P *= 0.009, odds ratio [OR] = 2.081, 95% confidence interval [CI] 1.198-3.615), dementia (*P *≤ 0.001, OR = 20.326, 95% CI 8.354-49.459), Parkinson's disease (*P *= 0.049, OR = 3.920, 95% CI 1.005-15.295), CKD (*P *= 0.018, OR = 2.542, 95% CI 1.172-5.512), AFIB (*P *= 0.017, OR = 1.863, 95% CI 1.118-3.103), HF (*P *= 0.002, OR = 2.411, 95% CI 1.389-4.185), and coronary artery disease (*P *= 0.004, OR = 2.434, 95% CI 1.324-4.475).

Conclusions: Among diseases independently associated with frailty, chronic diseases such as respiratory diseases, dementia, Parkinson's disease, CKD, and cardiovascular diseases (AFIB, HF, and coronary heart disease) have an important role. Recognizing the diseases that are highly related to frailty may contribute, by their optimal management, to delaying the progression or even reversing frailty in a large proportion of the elderly.

## Introduction

Over the past 100 years, advances in medical science have led to a doubling of life expectancy. Nowadays, 8.5% of the world's population is over 65 years old, and it is estimated that this figure will be 16.7% by 2050 [1]. Many health problems are associated with aging such as chronic diseases, infections, falls, and impaired cognitive function. People of the same chronological age may have different biological ages since aging is not synonymous with comorbidity and reduced functional capacity. Therefore, because age is not sufficient to describe the general condition of an older person, the term *frailty* has been increasingly used in recent years [2]. Frailty is not the same as aging, nor is it the same as disability and comorbidity. Comorbidity is a causative factor, whereas disability is a result of frailty [3]. So, frailty is defined as a state of increased vulnerability due to an age-dependent decline in the physiological reserves needed to maintain biological homeostasis [4].

Frailty is a clinical syndrome of older adults that leads to adverse health outcomes and an increased likelihood of hospitalization and mortality [5]. Cellular and systemic alterations, including sarcopenia, reduced dietary intake, and reduced physical activity, contribute to frailty [6]. Previous reports have associated frailty with the presence of various chronic diseases [6], and its relationship with cardiovascular diseases, in particular, appears to be bidirectional, with common pathophysiological mechanisms contributing to the progression of both conditions [7].

The identification of diseases directly related to frailty may have a dual benefit: first, better control of these diseases will result in an improvement in the level of frailty, and second, perhaps interventions that improve the level of frailty [8] may result in better control of these diseases. So, we conducted this study to enrich the existing literature with findings dealing with the relationship between frailty and chronic diseases.

This manuscript is a shortened version of a final thesis previously published in Greek by Andreas Kyvetos for his master’s degree at the National and Kapodistrian University of Athens. 

## Materials and methods

Study population and protocol

The study sample consisted of 457 consecutively admitted older patients who were hospitalized in the Second Department of Internal Medicine of the General and Oncological Hospital of Kifissia ‘’ Agioi Anargyroi’’ from September 2022 to June 2023. The inclusion criterion for the study was patients aged 65 years or older. Exclusion criteria included the onset of new acute diseases such as myocardial infarction (MI) and cerebrovascular accidents within the last six months, as well as a Clinical Frailty Scale (CFS) score above 8. By definition, a CFS score of 9 refers to persons with a shortened lifespan *who are not otherwise living with severe frailty.* The inclusion of such patients would have confused the results [9].

Definition of current illness

Medical history was obtained through interviews with the patient and caregiver and from the electronic prescribing system, following patient consent. The patient's chronic conditions that were treated continuously for the previous 3 months were recorded.

Definition of frailty

Recently published guidelines from the International Conference of Frailty and Sarcopenia Research (ICFSR) recommend the use of a validated and simple frailty screening tool [10]. The translated and validated CFS in the Greek language was used as the diagnostic instrument for frailty [11]. According to CFS, patients are classified into nine categories from 1 to 9, depending on the degree of frailty. Frail patients are those who have CFS > 4 [12]. The frailty level was calculated for the prehospital status of patients retrospectively since it can be used reliably before the onset of acute disease [13].

Covariates

Demographic data (age, sex, marital status, body weight, and body mass index), number of medications, and comorbidities using the Charlson comorbidity index [14] were recorded for each patient. Polypharmacy was defined as the use of five or more prescribed medications per day [15]. 

Ethical approval

The study was approved by the Institutional Ethical and Scientific Committee of General and Oncology Hospital of Kifissia “Agioi Anargyroi” (approval number 1663). All patients who participated completed a written consent form after being fully informed by the researchers.

Statistical analysis

All analyses were performed through the use of IBM SPSS Statistics for Windows, Version 22.0 (IBM Corp., Armonk, NY). Categorical data are expressed as counts and percentages. The normality of the continuous variable age was evaluated using the Shapiro-Wilk test. The distribution of age was not normal, and it was expressed as median and interquartile range (IQR). Differences in comorbidities between frail and non-frail patients were assessed by using the chi-square test. A *P*-value ≤ 0.05 was considered statistically significant. Comorbidities that differed statistically significantly between frail and non-frail patients were included in a separate binary logistic regression analysis, to identify the most important ones. Regarding the logistic regression model, the results are presented as odds ratios (OR), including a 95% confidence interval (CI).

## Results

A comprehensive collection of 457 patient samples was procured, revealing a balanced gender distribution with 231 individuals (50.5%) identified as male and 226 (49.5%) as female. The average age of the sampled population stood at 82, ranging from 75 to 89 years. Employing the Greek CFS, patients were stratified into two groups: those classified as frail, encompassing 277 patients (60.6%), with CFS scores ranging from 5 to 8, and those deemed non-frail, comprising 180 patients (39.4%), with CFS scores ranging from 1 to 4. For a comprehensive overview of the sample characteristics, refer to Table 1.

**Table 1:** Sample characteristics. Frail CFS 1-4 and non-frail CFS 5-8. CFS, Clinical Frailty Scale

Parameter	*N* = 457, *n *(%)	Frail, *n *(%)	Non-frail, *n *(%)
		277 (60.6 %)	180 (39.4%)
Gender			
Male	231 (50.5%)	125 (45.1%)	106 (58.9%)
Female	226 (49.5%)	152 (54.9%)	74 (41.1%)
Family status			
Married	212 (46.4%)	104 (37.5%)	108 (60%)
Unmarried	17 (3.4%)	9 (3.2%)	8 (4.4%)
Divorced	11 (2.4%)	3 (1.1%)	8 (4.4%)
Widowed	217 (47.5%)	161 (58.1%)	56 (31.1%)
Educational level			
Primary	273 (59.7%)	186 (67.1%)	87 (48.3%)
Secondary	106 (23.2%)	57 (20.6%)	49 (27.2%)
Technological education institution	51 (11.2%)	24 (8.7%)	27 (15%)
University	27 (5.9%)	10 (3.6%)	17 (9.4%)
Body weight			
Normal	227 (49.7%)	128 (46.2%)	99 (55%)
Lean	92 (20.1%)	65 (23.5%)	27 (15%)
Overweight	125 (27.4%)	76 (27.4%)	49 (27,2%)
Obese	13 (2.8%)	8 (2.9%)	5 (2.8%)
Age, median (IQR) (in years)	82 (75-89)	86 (80-90)	77.5 (71-83)

The prevalent chronic diseases documented included arterial hypertension affecting 276 patients (60.4%), followed by diabetes mellitus in 146 patients (31.9%), atrial fibrillation (AFIB) in 134 patients (29.3%), and heart failure (HF) in 115 patients (25.2%). The frequencies of all recorded diseases are enumerated in Table 2.

**Table 2:** Frequencies of all recorded diseases.

Chronic diseases	n	%
Arterial hypertension	276	60.4%
Diabetes mellitus	146	31.9%
Dyslipidemia	142	31.1%
Atrial fibrillation	134	29.3%
Heart failure	115	25.2%
Respiratory diseases	105	23%
Dementia	102	22.3%
Thyroid diseases	82	17.2%
Coronary heart disease (CHD)	77	16.8%
Depression	64	14%
Solid neoplasms	62	13.6%
Benign prostate hyperplasia	45	9.8%
Chronic kidney disease	45	9.8%
Cerebrovascular stroke	40	8.8%
Osteoporosis	39	8.5%
Parkinson's disease	34	7.4 %
Hematological malignancies	26	5.7%
Rheumatic-autoimmune diseases	22	4.8%
Anxiety disorder	22	4.8%
Gastroesophageal reflux disease	22	4.8%
Osteoarthritis	12	2.6%
Epilepsy	12	2.6%
Psychosis	11	2.4%
Vertigo	11	2.4%
Peripheral vascular disease	9	2.0%
Liver cirrhosis	9	2.0%
Glaucoma- ataract	8	1.8%
Deep vein thrombosis	5	1.1%
Multiple sclerosis	2	0.4%
Chronic hepatitis B	2	0.4%

Diseases with a prevalence below 5% (23 patients) were omitted from the statistical analysis due to their minimal occurrence. In the univariate analysis employing the chi-square method, frail patients exhibited a higher likelihood of presenting with respiratory diseases (*P *= 0.005, *χ*^2^ = 7.908), dementia (*P *< 0.001, *χ*^2 ^= 61.473), Parkinson's disease (*P *< 0.001, *χ*^2 ^= 14.343), chronic kidney disease (CKD, *P *= 0.031, *χ*^2 ^= 4.469), AFIB (*P *= 0.002, *χ*^2^ = 9.660), depression (*P *= 0.002, *χ*^2 ^= 8.560), vascular stroke (*P *= 0.051, *χ*^2 ^= 3.801), HF (*P *< 0.001, *χ*^2 ^= 14.559), and coronary heart disease (*P *= 0.008, *χ*^2 ^= 6.979) (Table 3).

**Table 3:** Association between comorbidities and frailty status of patients using chi-square. A value of *P* ≤ 0.05 was considered statistically significant.

Diseases		Frail		Non-frail		*P*-value
		n	%	n	%	
Respiratory diseases	Yes	76	16.6	29	6.3	0.005
	No	201	43.9	151	33	
Diabetes mellitus	Yes	90	19.6	56	12.2	0.757
	No	187	40.9	124	27.1	
Heart failure	Yes	87	19.0	28	6.1	<0.001
	No	190	41.5	152	33.2	
Rheumatic-autoimmune diseases	Yes	16	3.5	7	1.5	0.367
	No	261	57.1	173	37.8	
Atrial fibrillation	Yes	96	21.0	38	8.3	0.002
	No	181	39.6	142	31.0	
Osteoporosis	Yes	22	4.8	17	3.7	0.574
	No	255	55.7	163	35.6	
Arterial hypertension	Yes	160	35.0	116	36.3	0.154
	No	117	25.6	64	14.0	
Dyslipidemia	Yes	85	18.5	57	12.4	<0.001
	No	181	39.6	174	38.0	
Dementia	Yes	96	21.0	6	1.3	<0.001
	No	181	39.6	174	38.0	
Solid neoplasms	Yes	31	6.7	31	6.7	0.066
	No	246	53.8	149	32.6	
Parkinson's disease	Yes	31	6.7	3	0.6	<0.001
	No	246	53.8	177	38.7	
Depression	Yes	50	10.9	14	3.0	0.002
	No	227	49.6	166	36.3	
Cerebrovascular stroke	Yes	30	6.5	10	2.1	0.051
	No	247	54.0	170	37.1	
Ischemic heart disease	Yes	57	12.4	20	4.3	0.008
	No	220	48.1	160	35.0	
Thyroid diseases	Yes	55	12.0	27	5.9	0.186
	No	222	48.5	153	33.4	
Benign prostatic hyperplasia	Yes	28	6.1	17	3.7	0.816
	No	249	54.4	163	35.6	
Chronic kidney disease	Yes	34	7.4	11	2.4	0.031
	No	243	53.1	169	36.9	

Subsequently, a binomial regression analysis was conducted to assess the impact of statistically significant variables on the likelihood of frailty. The logistic regression model demonstrated statistical significance (*χ*^2^(10) = 142.324, *P* ≤ 0.001), explaining 36.2% of the variance in the probability of frailty (Nagelkerke R2) and correctly classifying 73.5% of cases. Notably, in the binomial regression analysis, several diseases exhibited significant associations with frailty, including respiratory diseases (*P *= 0.009, OR = 2.081, 95% CI 1.198-3.615), dementia (*P *≤ 0.001, OR = 20.326, 95% CI 8.354-49.459), Parkinson's disease (*P* = 0.049, OR = 3.920, 95% CI 1.005-15.295), CKD (*P* = 0.018, OR = 2.542, 95% CI 1.172-5.512), AFIB (*P* = 0.017, OR = 1.863, 95% CI 1.118-3.103), HF (*P* = 0.002, OR = 2.411, 95% CI 1.389-4.185), and coronary heart disease (*P* = 0.004, OR = 2.434, 95% CI 1.324-4.475) (Table 4).

**Table 4:** Summary of binary logistic regression analysis for the association between chronic diseases and frailty. A value of P ≤ 0.05 was considered statistically significant. B, coefficient estimate; SE, standard error; Wald, Wald statistic; Sig., significance level (*P*-value); Exp. (*B*), exponentiated coefficient (odds ratio); 95% CI for Exp. (*B*), 95% confidence interval for the odds ratio

Diseases	B	SE	Wald	Sig.	Exp. (*B*)	95% CI for Exp. (*B*)	
						Lower	Upper
Respiratory diseases	0.733	0.282	6.757	0.009	2.081	1.198	3.615
Heart failure	0.880	0.281	6.757	0.002	2.411	1.389	4.185
Atrial fibrillation	0.622	0.260	5.707	0.017	1.863	1.118	3.103
Dementia	3.012	0.454	44.0701	0.000	20.326	8.354	49.459
Solid neoplasms	-0.440	0.322	0.019	0.891	0.957	0.509	1.798
Parkinson's disease	1.366	0.695	3.868	0.049	3.920	1.005	15.295
Depression	0.682	0.368	3.440	0.064	1.978	0.962	4.065
Cerebrovascular stroke	0.319	0.451	0.498	0.480	1.375	0.568	3.331
Ischemic heart disease	0.890	0.311	8.200	0.004	2.434	1.324	4.475
Chronic kidney disease	0.933	0.395	5.582	0.018	2.542	1.172	5.512

## Discussion

To our knowledge, this study is the first in Greece to correlate all chronic diseases with the existence or not of frailty. The results of the study showed that the diseases that were statistically significantly associated with the presence of frailty are respiratory diseases, dementia, Parkinson's disease, CKD, and cardiovascular diseases (AFIB, HF, and coronary artery disease).

Previous studies have assessed the association between frailty and chronic respiratory diseases such as chronic obstructive pulmonary disease (COPD) and asthma [13,14]. Their findings have highlighted a strong association between these diseases and frailty [16]. Moreover, one in five patients with COPD is being diagnosed with frailty. The main factors that contributed to frailty in those patients were smoking, low physical activity, repeated exacerbations, hospitalizations, poor nutrition, and polypharmacy. In addition, frailty is important to diagnose in these patients as it is an independent predictor for hospital admissions and mortality [17].

The association between frailty and neurological diseases is well known, especially for dementia. It has been recognized that a higher level of frailty leads to an increased likelihood of developing dementia, and therefore, frailty has been suggested as an important modifiable factor in protecting against the onset of dementia [18]. Likewise, a strong association between frailty and Parkinson's disease has been demonstrated by other studies [19].

In addition, and in line with our results, CKD has been associated with frailty in previous studies. Especially for patients on hemodialysis, the incidence of frailty is up to 60%. The high incidence of physical frailty in CKD patients is explained in the context of anorexia which leads to reduced energy intake and sarcopenia [20].

Regarding the relationship between frailty and AFIB, the various studies conducted have shown conflicting results. AFIB is the most common persistent arrhythmia in the elderly, with an incidence of 23% in people over 84 years of age [21]. A recently published cohort study from AHA by Orkaby et al. did not show a statistically significant relationship between frailty and AFIB [22]. In contrast, another study by Hang et al. showed a statistically significant relationship between frailty and first-onset AFIB in patients with arterial hypertension [23]. The degree of frailty in the elderly and the development of AFIB share common pathophysiological mechanisms such as a strong inflammatory response, poor immune function, and neurological damage. Furthermore, frailty itself affects the autonomic nervous system, which, in turn, has an important role in both the development and maintenance of AFIB [24,25]. In addition, AFIB has been associated with cerebral ischemia, cognitive impairment, and dementia [26]. The presence of AFIB in the Health Aging and Body Composition study showed that it had a negative impact on the physical performance of individuals [27].

There is particular interest in the relationship between frailty and HF since chronic inflammatory response and sarcopenia are common features both in frailty and HF. Moreover, frailty has been reported as a marker of poor prognosis for patients with HF [28]. Their relationship is bidirectional, as many studies have shown that frailty increases the likelihood of acute HF, and vice versa, HF worsens the degree of frailty [29-32]. The incidence of frailty in patients with HF varies by the studied population (inpatients-community dwelling) and the diagnostic tools used to detect it. In a recent review by Denfeld et al., the incidence of frailty in patients with HF was 44.5% [33]. At the same time, frailty was more common in patients with HF with preserved ejection fraction (HFpEF) than in patients with reduced EF (HFrEF). This is probably related to the fact that patients with HFpEF suffered from more comorbidities compared to those with HFrEF [28]. In addition, regarding outcome, frail patients with HF have an increased risk of mortality and hospitalization compared to non-frail patients [34-39].

The relationship between frailty and coronary artery disease is also bidirectional. More specifically, on one hand, coronary artery disease increases the likelihood of frailty, and on the other hand, frailty increases the likelihood of coronary artery disease [40-42]. A systematic review concluded that one-fifth of patients with frailty have ischemic heart disease (IHD), whereas one-fifth of patients with IHD are frail. Atherosclerosis leading to IHD is now considered a systemic inflammatory disease. In particular, a chronic increase in pro-inflammatory cytokines such as interleukin-6 (IL-6), IL-1β, IL-17, and tumor necrosis factor-alpha (TNF-α) characterizes IHD. Similarly, low-grade inflammation and immune activation play an important role in the pathophysiology of frailty. Inflammation leads to detrimental effects on skeletal muscle since it promotes cell apoptosis, leading to sarcopenia. Additionally, in chronic inflammation, the neuroendocrine system is also affected, leading to an increased likelihood of cardiovascular disease, loss of appetite, weight loss, and metabolic disorders, which result in reduced muscle mass and strength. From all of the above, it is concluded that frailty and IHD share a common pathophysiological mechanism with chronic inflammation, which explains the biological relationship between these two conditions [43]. Frailty has been associated in many studies with poor prognosis in acute coronary syndromes. A recent systematic review and meta-analysis of 8,554 patients showed that the presence of frailty is significantly associated with mortality in both patients presenting with ST-segment elevation myocardial infarction (STEMI) and non-STEMI [44].

Our study did not show a statistically significant relationship between arterial hypertension and frailty. Although, the relationship between arterial hypertension and frailty is uncertain, as shown in a meta-analysis by Vetrano et al., arterial hypertension is common in people with frailty, with an incidence of 72%, whereas the incidence of frailty in patients with arterial hypertension is 14% [45].

This study has some limitations. First, it was a single-center, single-time point study in hospitalized patients. Since the study sample was limited to hospitalized patients, findings on the prevalence of frailty and chronic diseases, and other features of the study sample cannot be applied to the entire community. Second, the study's cross-sectional methodology precludes drawing conclusions about causality.

## Conclusions

Identifying the diseases directly related to frailty may have a dual benefit: first, better control of these diseases will result in an improvement in the level of frailty, and second, perhaps with interventions that improve the level of frailty, better control of these diseases can be achieved. After all, frailty itself is a potentially treatable condition through interventions such as physical, pharmaceutical, dietary, and psychological that could prevent, delay, or reverse it. Future longitudinal studies could identify if delaying or reversing frailty, as a part of a holistic treatment approach, might contribute to the optimal management of these chronic diseases. Furthermore, they could investigate whether optimizing the management of these diseases might contribute to delay or reverse frailty.
